# Effects of light intensity and plant growth regulators on callus proliferation and shoot regeneration in the ornamental succulent *Haworthia*

**DOI:** 10.1186/s40529-019-0257-y

**Published:** 2019-07-02

**Authors:** Yen-Ming Chen, Jian-Zhi Huang, Ting-Wen Hou, I-Chun Pan

**Affiliations:** 1 Department of Horticulture, National Chung Hsing University, No. 145, Xingda Road, Taichung, 402 Taiwan; 2 Department of Plant Industry, National Pingtung University of Science and Technology, No. 1, Shuefu Road, Neipu, 912 Pingtung Taiwan; 3 Department of Agricultural Chemistry, College of Bioresources and Agriculture, National Taiwan University, No. 1, Sec. 4, Roosevelt Rd., Taipei, 106 Taiwan

**Keywords:** *Haworthia*, Inflorescence explants, 6-Benzylaminopurine, α-Naphthalene acetic acid, Callus, Shoot, Light intensity

## Abstract

**Background:**

*Haworthia* are desert succulents belonging to the Asphodelaceae family. *Haworthia* species are cultivated commercially as ornamentals and some rare species are quite valuable at retail market but growth slowly and difficult to propagation. However, an efficient micropropagation protocol was remained insufficient.

**Results:**

The organogenic cultures obtained from inflorescence explants were cultured on Murashige and Skoog (MS) medium supplemented with various combinations of 6-benzylaminopurine (BA) and α-naphthalene acetic acid (NAA) under a light intensity of 10 μmol m^−2^ s^−1^ or 45 μmol m^−2^ s^−1^. The highest callus proliferation index (93.15%) with 1.0 mg L^−1^ BA + 0.1 mg L^−1^ NAA under a light intensity of 10 μmol m^−2^ s^−1^. The best shoot proliferation rates were on media with either 1 mg L^−1^ BA + 0–0.4 mg L^−1^ NAA (65.57–81.01%) under a light intensity of 45 μmol m^−2^ s^−1^. The highest root length (15.57 mm) and the highest rooting frequency (17 roots per shoot) were obtained when adventitious shoots were inoculated on MS medium with 0.4 mg L^−1^ NAA + 0.4 mg L^−1^ IBA. The survival rate of the transplanted plantlets was about 100%. The efficient micropropagation protocol proliferated *Haworthia* regenerate plants from inflorescence within 11 weeks.

**Conclusions:**

The present study determined the best combination of light intensity and plant growth regulators (PGRs) for improved organogenesis of *Haworthia* during propagation by tissue culture. This optimized protocol showed light intensity is an important factor for efficient callus or shoot regeneration. These results indicate that it will be useful to optimize the light conditions for future commercial cultivation, germplasm conservation, genetic engineering and molecular biology research of this ornamental plant.

## Background

Plants in the genus *Haworthia* are perennial, monocotyledonous, desert succulents belonging to the Asphodelaceae family. Species in the genus are distributed in Southeast Africa, Namibia and Madagascar. *Haworthia* species are cultivated commercially as ornamentals, while rare species are of interest to collectors (Bayer 1982). Some rare species are quite valuable, but growth slowly and difficult to propagation. In the retail market, the price of *Haworthia* specimen are determined by their population, shapes of leaf window, leaf stripe, or leaf variegation.

Traditionally, *Haworthia* species are propagated from seed, leaf cutting, crown division, or the propagules produced on the inflorescence (Pilbeam 1983), all of require time. In recent years, micropropagation has been shown to have a vast potential for quickening the propagation time for species in many succulent genera, including Notocactus (Seol et al. 2008), Cotyledon (Kumari et al. 2016), Pelecyphora (Badalamenti et al. 2016), Kalanchoe (Jaiswal and Sawhney 2006) and Aloe (Bairu et al. 2007). Micropropagation has also been used to aid in the reproduction of some self-incompatible *Haworthia* species (Mycock et al. 1997), through inducing regeneration from leaves (Beyl and Sharma 1983; Rogers 1993), inflorescences (Kaul and Sabharwal 1972; Majumdar 1970a) and ovary walls (Majumdar 1970b).

Micropropagation efficiency or success rate is affected by various factors, such as explant type, nutrients, plant growth regulators and other additives, temperature, and light intensity and duration. *Haworthia* callus have been induced from leaf segments, inflorescence segments, or flower buds (Kaul and Sabharwal 1972; Ogihara 1978). The in vitro growth characteristics of some *Haworthia* species have been examined in response to different medium supplements, such as auxins (IAA, NAA, 2,4-D) (Kaul and Sabharwal 1972; Ogihara 1978, 1979), cytokinins (BA, kinetin, zeatin) (Kaul and Sabharwal 1972; Ogihara 1979; Richwine et al. 1995; Rogers 1993), defoliants (thidiazuron) (Liu et al. 2017), casein hydrolysate (Kaul and Sabharwal 1972), coconut milk (Kaul and Sabharwal 1972) and inositol (Kaul and Sabharwal 1975). The effect of light intensity on in vitro shoot proliferation of *Haworthia* species has not been reported.

Light is one of the important environmental factors that controls plant growth, development, morphogenesis, metabolism and chlorophyll content in plant cell, tissue and organ cultures (Dou et al. 2017). Light intensity and light source are important parameters that influence shoot regeneration, fresh weight, and secondary metabolite biosynthesis during micropropagation in a variety of crops, including Persian shallot (Farhadi et al. 2017), cauliflower (Kumar et al. 1993), date palm (Meziani et al. 2015) and *Cistanche* (Ouyang et al. 2003). Light can also influence the efficacy of plant growth regulators (PGRs) as well as adjustment of endogenous hormone levels. Recent studies have resolved the light regulation of gibberellin and auxin metabolism (García-Martinez and Gil 2001; Halliday et al. 2009; Stavang et al. 2007).

To establish a highly productive and rapid in vitro plant regeneration system for *Haworthia* spp., the inflorescence of the *Haworthia* cultivar ‘Sansenjyu’ were excised for micropropagation. Besides growth regulators, light intensity was another important factor to improve the regeneration efficiency. An efficient regeneration system will be useful for future commercial cultivation and biology research of this ornamental crop, and be a foundation for regeneration system of succulent plants.

## Methods

### Plant material, primary culture medium and conditions

Soil-grown, adult *Haworthia* ‘Sansenjyu’ (*H. obtusa* ×* H. comptoniana*) plants were obtained from Guoguang flower market in Taichung Taiwan. The plants were maintained at 25/30 °C night/day temperatures in a greenhouse under a 12-h/12-h photoperiod. Inflorescences were excised from healthy plants and were surface-sterilized first with 70% ethanol for 60 s and then with 0.6% sodium hypochlorite containing 1 drop of Tween 20 per 100 ml solution for 20 min. The explants were then rinsed five times with sterile, distilled water. The disinfected pedicels were then cut into 5-mm segments and inoculated on callus induction medium, which consisted of Murashige and Skoog (MS) medium (Murashige and Skoog 1962) supplemented with 30 g L^−1^ sucrose, 0.1 g L^−1^ myo-inositol, 2 mg L^−1^ 6-benzylaminopurine (BA), 0.2 mg L^−1^ α-naphthalene acetic acid (NAA), and solidified with 0.8% agar. The chemicals or plant growth regulators were analytical grade (Duchefa Biochemie). All media were adjusted to pH 5.7 with 1 N NaOH or 1 N HCl before autoclaving at 121 °C and 103 kPa for 20 min. The cultures were maintained at a temperature of 26 °C and a light intensity of 45 μmol m^−2^ s^−1^ provided by cool white fluorescent light with a 16-h photoperiod. The percentage of explants producing callus tissue was recorded after 8 weeks. Calli were then used for callus propagation and plant regeneration.

### Callus propagation, adventitious shoot regeneration and light conditions

The callus that developed from the inflorescence explants was separated into small pieces (≈ 50 mg) and transferred onto MS basal medium supplemented with different concentrations of BA (0, 1, 2, or 4 mg L^−1^) and NAA (0, 0.1, 0.2, or 0.4 mg L^−1^) for callus propagation and adventitious shoot initiation. Cultures were maintained in light at an intensity of 10 μmol m^−2^ s^−1^ or 45 μmol m^−2^ s^−1^ at 26 °C under a 16-h photoperiod. After 8 weeks, the weight of the callus, the number of adventitious shoots, and the browning rate of the callus were measured. Each experiment was repeated five times, each using 15 replicates (i.e., a total of 75 explants per treatment).

### Root induction and plantlet acclimatization

For root induction, excised shoots (2–3 cm) were transferred to MS basal medium supplemented with different concentrations of NAA (0, 0.1, 0.2, or 0.4 mg L^−1^) or indole-3-butyric acid (IBA) (0, 0.1, 0.2, or 0.4 mg L^−1^). The cultures were maintained at a temperature of 26 °C and a light intensity of 45 μmol m^−2^ s^−1^. The root length and number of shoots were measured after 4 weeks. Each experiment was repeated five times. For plant acclimatization, rooted plantlets were carefully transferred to 9-cm pots containing a 1:1:1 mixture of perlite, vermiculite and perlite and placed in a growth chamber under a 16-h photoperiod at 90% humidity under a day/night temperature regime of 26 °C.

### Statistical analysis

The experimental data were analyzed statistically by SAS 9.4 (Statistics Analysis System) software. Data on the effects of the hormone treatments at various concentrations and combinations were tested using least significant difference (LSD).

## Results

### Callus induction and morphogenesis in vitro

**Fig. 1:**
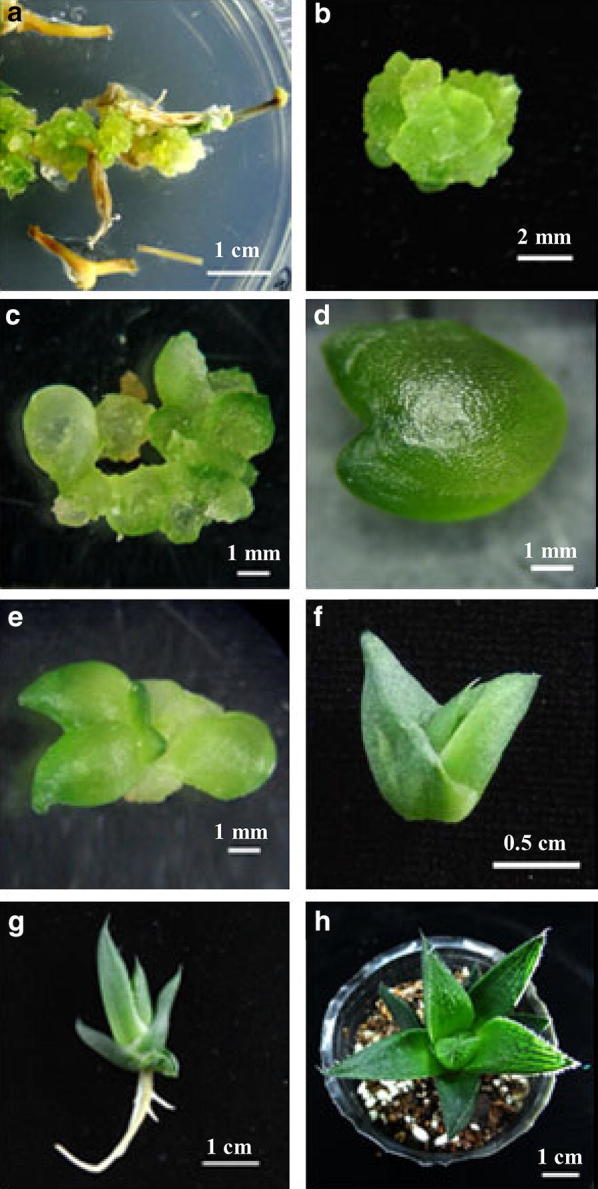
Somatic embryogenesis in *Haworthia* ‘Sansenjyu’ calli. **a** Calli initiation from inflorescences, **b** embryogenic calli, **c** globular stage somatic embryo, **d** heart stage somatic embryo, **e** shoot formation, **f** plantlet, **g** root formation, and **h** acclimated plant

The inflorescence of *H. obtusa* ×* comptoniana* ‘Sansenjyu’ were easy to collect, without harm to the mother plants, and to sterilize. On the basic callus induction medium, friable calli started to form at the cut edges of the inflorescence after 2 weeks (Fig. 1a). The green and compact embryogenic calli were observed on most of the explants within 3 weeks (Fig. 1b). Numerous globular stage and heart stage somatic embryos were visible all around the surface of the embryogenic callus after 4 weeks (Fig. 1c, d). Shoot differentiation and formation began after 5 weeks (Fig. 1e), and adventitious shoots were observed with normal green leaves after 7 weeks (Fig. 1f). Roots were formed 4 weeks after explants transferred to root induction medium (Fig. 1g), and grown in a perlite, vermiculite and soil mixture (Fig. 1h).

### Effect of growth regulators and light intensity on callus proliferation

**Fig. 2:**
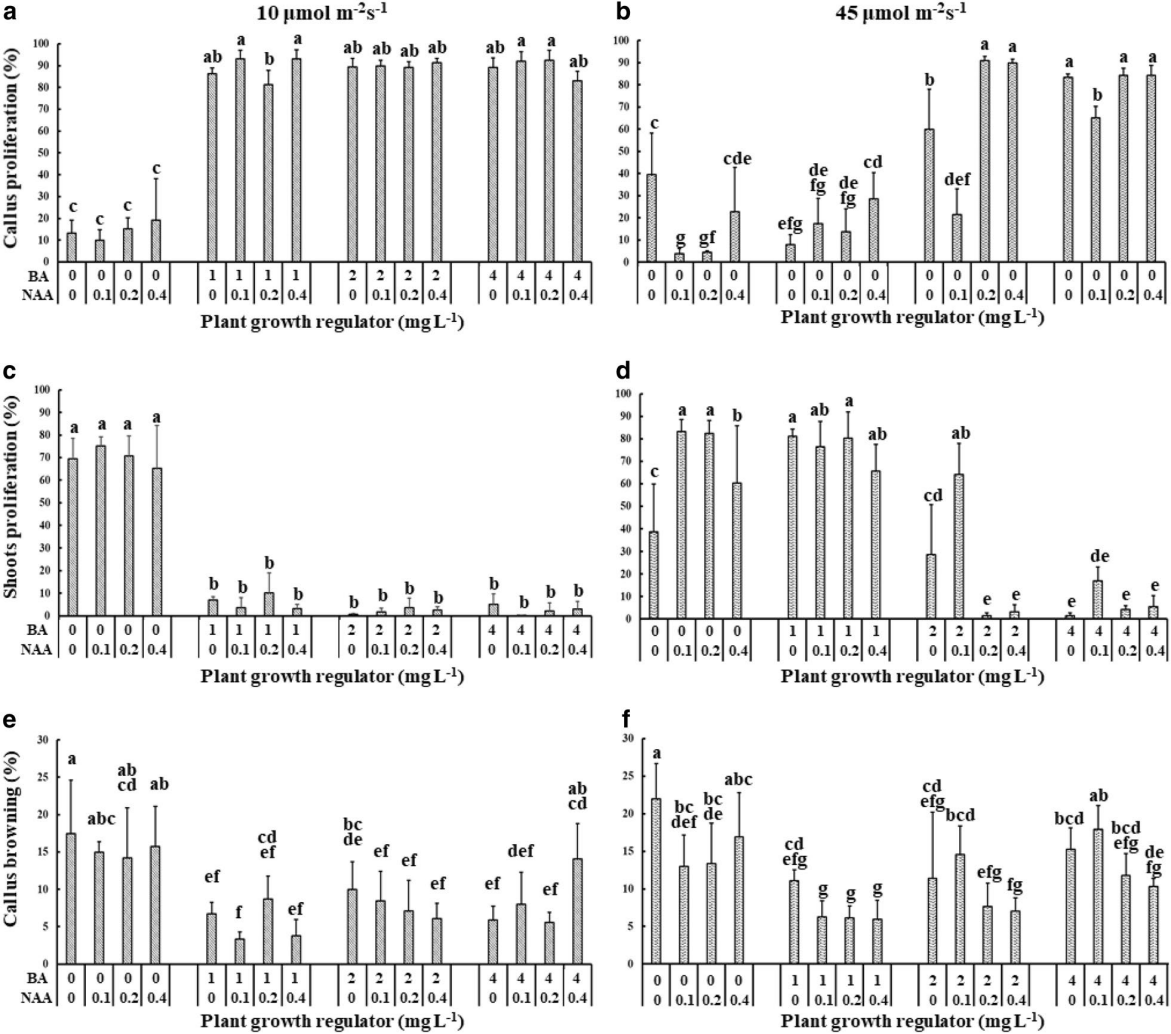
Effect of light intensity on in vitro proliferation of *Haworthia* ‘Sansenjyu’. The percentage of callus proliferation (**a**, **b**), shoot proliferation (**c**, **d**) and callus browning (**e**, **f**) under 10 μmol m^−2^ s^−1^ or 45 μmol m^−2^ s^−1^ light intensities

A total of 16 different medium combinations were tested under two different light intensities. The highest rates of callus proliferation were recorded when the initial callus pieces were placed on media containing 1 to 4 mg L^−1^ BA + 0 to 0.4 mg L^−1^ NAA under a light intensity of 10 μmol m^−2^ s^−1^. Under these conditions, the callus proliferation rate ranged from 81.22 to 93.15% (Fig. 2a). The lowest callus proliferation rates were observed on media containing 1 mg L^−1^ BA + 0 to 0.4 mg L^−1^ NAA media under a light intensity of 45 μmol m^−2^ s^−1^ (7.9 to 28.48%; Fig. 2b). Under the higher light intensity, callus proliferated better on medium containing 2 to 4 mg L^−1^ BA + 0 to 0.4 mg L^−1^ NAA, except on media containing 2 mg L^−1^ BA + 0.1 mg L^−1^ NAA (Fig. 2b). The results showed that a light intensity of 10 μmol m^−2^ s^−1^ was optimal for attaining proliferated callus and is more forgiving across a range of PGR combinations. The most cost-effective PGR combination with a high proliferation rate was 1 mg L^−1^ BA + 0.1 mg L^−1^ NAA.

### Effect of growth regulators and light intensity on adventitious shoot initiation

Shoot proliferation ratios also varied with light intensity. Under 10 μmol m^−2^ s^−1^ light intensity, shoot proliferation was better on medium without BA, and ranged from 65.14 to 75.11%. The shoot proliferation rate was lower than 4.99% when the medium contained 1 mg L^−1^ or more BA (Fig. 1c). Under 45 μmol m^−2^ s^−1^ light intensity, shoot proliferation showed relatively higher rates on 0 to 1 mg L^−1^ BA + 0 to 0.4 mg L^−1^ NAA medium, reaching the highest rate of 83.15%. Shoot proliferation decreased as the BA concentration increased (Fig. 1d). Our results demonstrated that higher light intensity caused a slight increase in the shoot proliferation.

The browning rate of the callus ranged from 3 to 22%. The highest rate of browning occurred on media containing 0 mg L^−1^ BA + 0 to 0.4 mg L^−1^ NAA under both light intensities (Fig. 2e, f). This further showed that BA is essential in callus medium for reducing callus browning. The callus browning rates were higher under the stronger light intensity (Fig. 2e, f). Our experiment proved that 10 μmol m^−2^ s^−1^ is the optimal incubation light intensity for attaining fully proliferated callus.

### Effect of auxins on rooting and acclimatization

**Fig. 3:**
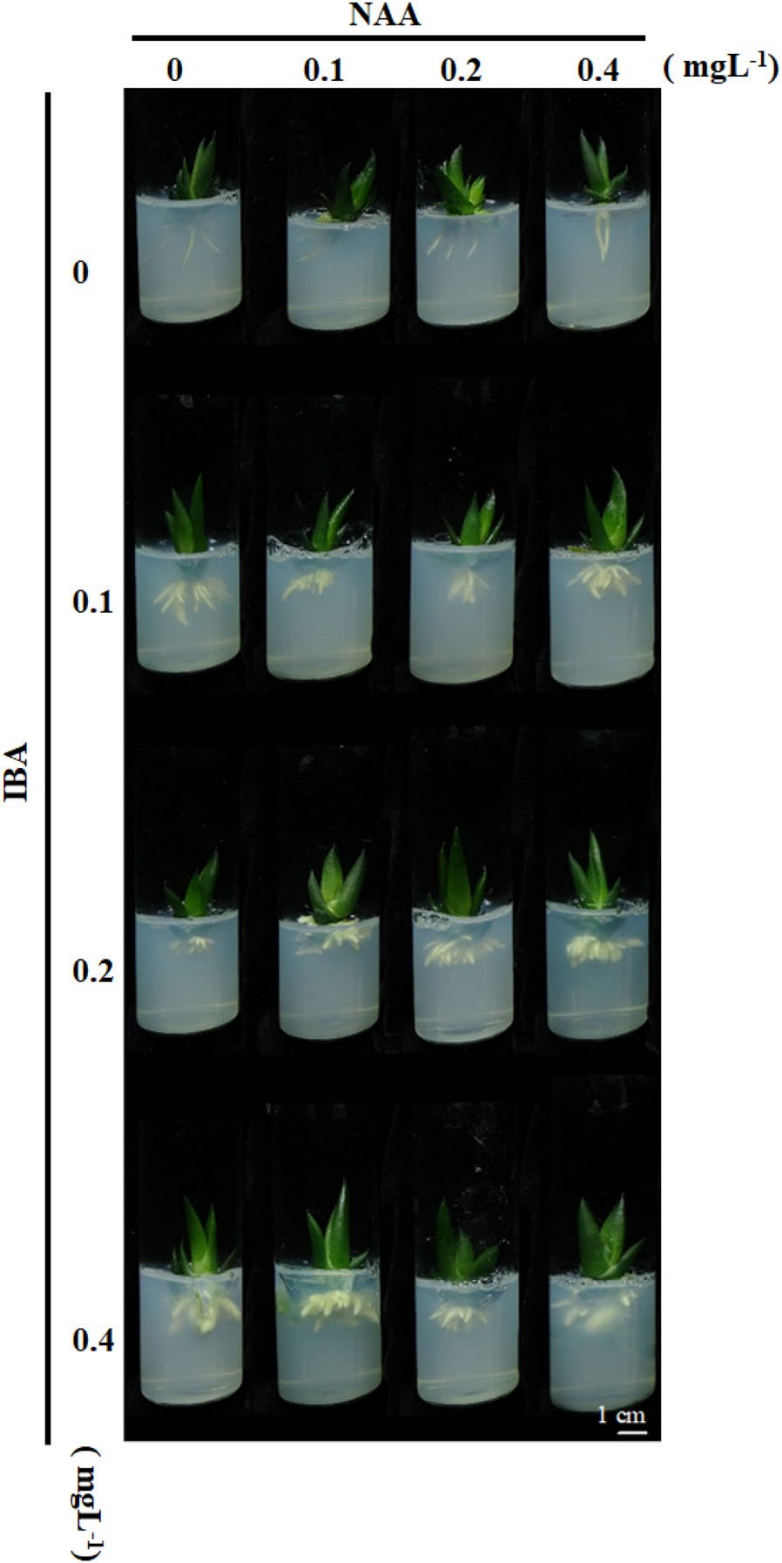
*Haworthia* ‘Sansenjyu’ root formation during cultivation on different concentrations of NAA and IBA for 4 weeks. MS medium contained combinations of 0, 0.1, 0.2, or 0.4 mg L^−1^ NAA and 0, 0.1, 0.2, or 0.4 mg L^−1^ IBA

**Fig. 4:**
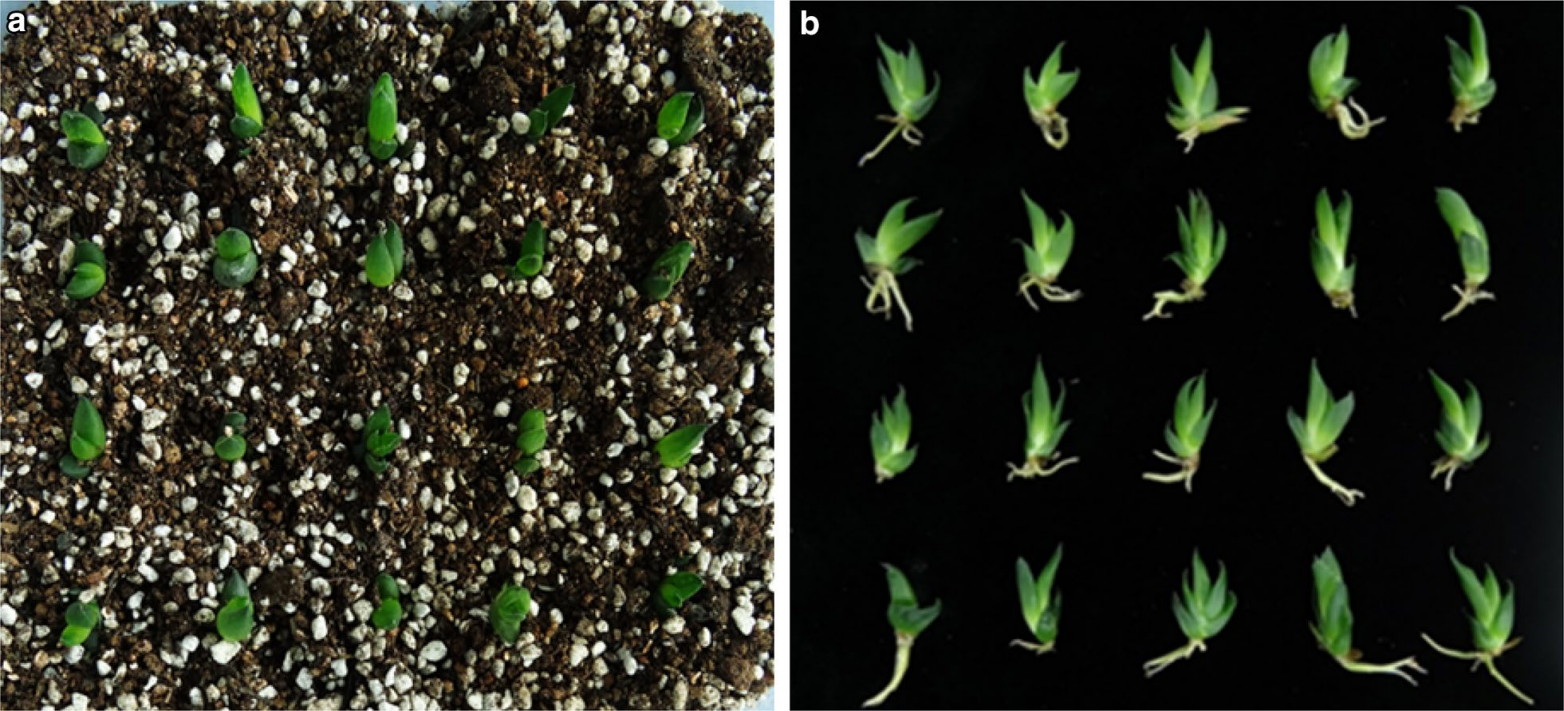
Haworthia ‘Sansenjyu’ plantlets (**a**) grown in a vermiculite, perlite and soil mixture under greenhouse conditions after 4 weeks, and (**b**) the root regeneration

Well-developed, regenerated individual *Haworthia* shoots (2 to 3 cm in height) from the multiplication stage were excised from the callus and transferred to root induction medium. Within 4 weeks, all shoots developed roots on MS media with or without plant growth regulators (Fig. 3). Out of the different IBA + NAA concentrations tested, the longest roots (15.57 mm) and highest rooting frequency (17 roots per shoot) were obtained after placing the shoots on MS medium supplemented with 0.4 mg L^−^ NAA + 0.4 mg L^−1^ IBA (Table 1 and 2). In general, root numbers increased with increasing NAA concentration (Table 2). The well-rooted plantlets were acclimated in a 1:1:1 (v/v) vermiculite: perlite: soil growing media under greenhouse conditions, with a 100% survival rate (Fig. 4a). The ex vitro rooting and hardening process was achieved concurrently with 100% success in the greenhouse (Fig. 4b).

**Table 1:** *Haworthia* ‘Sansenjyu’ root length after cultivation in MS medium with different concentrations of NAA and IBA for 4 weeks

NAA (mg L^−1^)	IBA (mg L^−1^)	Root length (mm)*
0	0	16.87 ± 5.65^abcd^
0	0.1	14.37 ± 2.3^abcdef^
0	0.2	11.32 ± 1.58^bcdefghij^
0	0.4	10.17 ± 2.1^defghij^
0.1	0	10.52 ± 3.85^cdefghij^
0.1	0.1	11.95 ± 2.65^abcdefghij^
0.1	0.2	11.43 ± 2.02^bcdefghij^
0.1	0.4	7.09 ± 0.82^ghij^
0.2	0	5.74 ± 0.5^ij^
0.2	0.1	12.33 ± 1.72^abcdefghi^
0.2	0.2	9.68 ± 1.34^efghij^
0.2	0.4	14.37 ± 1.44^abcdef^
0.4	0	13.27 ± 5.29^abcdefgh^
0.4	0.1	14.17 ± 1.12^abcdef^
0.4	0.2	10.72 ± 1.31^bcdefghij^
0.4	0.4	15.57 ± 4.36^abcde^

MS medium contained combinations of 0, 0.1, 0.2, or 0.4 mg L^−1^ NAA and 0, 0.1, 0.2, or 0.4 mg L^−1^ IBA. n = 5

*Different letters in each column indicate significant differences at *P* < 0.05. Data were analyzed with LSD using the SAS 9.4 program

**Table 2:** *Haworthia* ‘Sansenjyu’ root formation after cultivation on MS medium with different concentrations of NAA and IBA for 4 weeks

NAA (mg L^−1^)	IBA (mg L^−1^)	Root number*
0	0	3.00 ± 0.82^ijk^
0	0.1	2.00 ± 0.82^jk^
0	0.2	5.33 ± 0.94^fghijk^
0	0.4	1.67 ± 0.47^jk^
0.1	0	11.67 ± 2.05^abcde^
0.1	0.1	9.67 ± 3.09^cdefg^
0.1	0.2	8.33 ± 2.05^defghi^
0.1	0.4	5.67 ± 2.87^fghijk^
0.2	0	10.00 ± 3.56^cdefg^
0.2	0.1	14.67 ± 1.70^abc^
0.2	0.2	13.67 ± 3.40^abcd^
0.2	0.4	13.67 ± 4.50^abcd^
0.4	0	9.00 ± 3.74^cdefgh^
0.4	0.1	14.67 ± 2.87^abc^
0.4	0.2	16.67 ± 4.78^ab^
0.4	0.4	17.00 ± 4.32^a^

MS medium contained a combination of 0, 0.1, 0.2, or 0.4 mg L^−1^ NAA and 0, 0.1, 0.2, or 0.4 mg L^−1^. n = 5

*Different letters in each column indicate significant differences at *P* < 0.05. Data were analyzed with LSD using the SAS 9.4 program

## Discussion

The use of micropropagation for the improvement and propagation of *Haworthia* species has received little research attention. In plants, growth regulators play essential roles in controlling the growth and development. Supplementation of tissue culture media with some plant growth regulators is essential. Auxins are the main hormones responsible for stem elongation, phototropism, vascular tissue differentiation, and cell expansion in plant developmental processes and are sensitive to higher light intensity (Vanneste and Friml 2009). Cytokinins are required, in concert with auxin, for cell division in a wide variety of plant tissue cultures (D’Agostino and Kieber 1999; Dewitte et al. 1999). Cytokinins can promote stem elongation in light-grown plants (Lin and Cheng 1997; Smets et al. 2005). During tissue culture, factors including auxins, cytokinins, light and temperature influence callus induction, and at suboptimal concentrations and combinations can cause callus browning and necrosis (Afshari et al. 2011; Dou et al. 2017; Ikeuchi et al. 2013).

The present study describes the development of an efficient and reliable system for both callus and shoot proliferation from *Haworthia* explants derived from inflorescence. For a reliable proliferation system, both efficient and specific induction of callus amplification and shoot regeneration are essential. The combinations of 1 to 4 mg L^−1^ BA and 0 to 0.4 mg L^−1^ NAA under a light intensity of 10 μmol m^−2^ s^−1^ were found to be effective for callus proliferation (Fig. 2a). The reason for this enhanced callus proliferation may lay in the fact that natural auxin levels increase under lower light intensity or darkness (Chory et al. 1994). Higher BA concentrations were necessary for better callus proliferation under a higher light intensity (Fig. 2b).

An increased rate of callus browning was observed under the higher light intensity (Fig. 2e, f). Callus browning is caused by poly-phenolic compounds present when the explants were wounded and the enzymatic browning reaction of phenolic compounds, oxidized by polyphenol oxidase, peroxidise or exposure to air (Taranto et al. 2017). The oxidation process of phenolic compounds is enhanced by light, (Taranto et al. 2017). Therefore, the inflorescence-derived callus showed higher browning rate and darker browning color under a higher light intensity.

An efficient adventitious shoot regeneration system from direct or indirect organogenesis is an essential method for producing a large number of elite genotypes and avoiding somaclones. Multiple adventitious shoots were proliferated from calli under a higher light intensity (Fig. 2d). Light intensity or quality-dependent changes in plant physiology and morphogenesis are regulated by plant hormones (Afshari et al. 2011; Dietz 2015; Kissoudis et al. 2017). This result may be due to the interaction between light intensity, endogenous auxin and endogenous cytokinins, which directly or indirectly affect shoot proliferation.

In general, root induction can be initiated by adding auxins such as NAA, IBA,

and IAA (Sauer et al. 2013). Previous studies have reported the auxin requirements for *Haworthia* species such as *H. turgida* (Liu et al. 2017), *H. retusa* (Kim et al. 2018; Suzanne 1993), *H. attenuata* (Richwine et al. 1995) and *H. turgida* var. although the proliferation ability (Majumdar 1970b) for inducing adventitious root formation. A significantly higher rate of rooting (17 roots per explant; with an average root length of 15.57 mm) occurred in this study by using MS medium supplemented with 0.4 mg L^−1^ NAA + 0.4 mg L^−1^ IBA (Table 1 and Table 2). Root thickness increased with increasing IBA concentration (Fig. 3). However, in our experiments, plantlets could grow well after transfer to the greenhouse even if they had only a few roots.

A proliferation system for *Haworthia planifolia* var. *setulifera* (Albany Division) had been established from callus induced from leaf tissue and subsequent shoot and root differentiation. The calli were induced from leaf segments after 16 weeks, and the regenerated plants were obtained around 39 weeks later (Wessels et al. 1976). The inflorescence is another widely used tissue for micropropagation, and similar observations have been reported with inflorescences for several plants, e.g. Aranda orchid (Goh and Wong 1990), *Elaeis* (Jayanthi et al. 2015), *Aloe* (Rathore et al. 2011), *Musa* AAA (Pérez-Hernández and Rosell-García 2008), *Amaranthus* (Arya et al. 1993) and *Spathiphyllum* (Fonnesbech and Fonnesbech 1979). In this research, rapid proliferation, within 11 weeks, was achieved using inflorescence-derived calli and by optimizing plant growth regulators and light intensity for the *Haworthia* cultivar ‘Sansenjyu’ (*H. obtusa* × *comptoniana*). The increased multiplication rate and cost-effective, easy acclimatization process make this protocol highly advantageous. This method could be tried on different *Haworthia* species, although the proliferation ability and regeneration speed of different selections could be various and should be examined individually. Our data indicated that light intensity is an important factor in determining micropropagation efficiency. Furthermore, these results could promote research on the physiology, genetic engineering, and molecular biology of the *Haworthia* genus.

## Data Availability

Not applicable.
